# Specific and reliable detection of Myosin 1C isoform A by RTqPCR in prostate cancer cells

**DOI:** 10.7717/peerj.5970

**Published:** 2018-11-20

**Authors:** Aleena A. Saidova, Daria M. Potashnikova, Anna V. Tvorogova, Ivan V. Maly, Wilma A. Hofmann, Ivan A. Vorobjev

**Affiliations:** 1 Biological Faculty, M.V. Lomonosov Moscow State University, Moscow, Russia; 2 Laboratory of Atherothrombosis, Moscow State University of Medicine and Dentistry, Moscow, Russia; 3 A.N. Belozersky Institute of Physico-Chemical Biology, M.V. Lomonosov Moscow State University, Moscow, Russia; 4 Department of Physiology and Biophysics, Jacobs School of Medicine and Biomedical Sciences, State University of New York at Buffalo, Buffalo, United States of America; 5 Department of Biology, School of Science and Technology, Nazarbayev University, Astana, Kazakhstan

**Keywords:** Prostate cancer, Myosin 1C A isoform, RT qPCR

## Abstract

**Background:**

Prostate cancer (PC) diagnostics and treatment often present a challenging task due to cancer subtype heterogeneity and differential disease progression in patient subgroups. Hence, the critical issue is finding a reliable and sensitive diagnostic and prognostic PC marker, especially for cases of biopsies with low percentages of cancer cells. Isoform A of myosin 1C was shown to be expressed in PC cells and responsible for their invasive properties, however, its feasibility for diagnostic purposes remains to be elucidated.

**Methods:**

To verify the role of myosin 1C isoform A mRNA expression as a putative prostate cancer marker we performed RT qPCR normalized by three reference genes (*GAPDH, YWHAZ, HPRT1*) on PC3, RWPE-1, LNCaP and 22Rv1 cell lines. Myosin 1C isoform A detection specificity was confirmed by immunofluorescence staining, cancer and non-cancer prostate cell lines were immunophenotyped by flow cytometry.

**Results:**

Median normalized mRNA expression level of myosin 1C isoform A in PC cells (PC3 and 22Rv1) is two orders of magnitude higher compared to RWPE-1 cells, which functionally correspond to benign prostate cells. Myosin 1C isoform A expression allows PC cell detection even at a dilution ratio of 1:1000 cancer to non-cancer cells. At the protein level, the mean fluorescence intensity of myosin 1C isoform A staining in PC3 nuclei was only twice as high as in RWPE-1, while the immunophenotypes of both cell lines were similar (CD44+/CD90-/CD133-/CD57-/CD24+-).

**Conclusions:**

We report a distinct difference in myosin 1C isoform A mRNA levels in malignant (PC3) and benign (RWPE-1) prostate cell lines and suggest a combination of three reference genes for accurate data normalization. For the first time we provide an immunophenotype comparison of RWPE-1 and PC3 cells and demonstrate that RT qPCR analysis of *MYO 1C A* using appropriate reference genes is sufficient for PC detection even in low-abundance cancer specimens.

## Introduction

Prostate cancer is one of the most frequent cancer types in men and the second most common factor of cancer-related morbidity in the Western world (Siegel, Miller & Jemal, 2015). Usually, prostate cancer occurs in an indolent form, however some subtypes can be aggressive and require immediate treatment. Sensitive diagnostic tools are critical for early metastatic prostate cancer detection, as early diagnosis ensures better clinical outcomes (Kawachi et al., 2007; Swanson et al., 2006). Multiple screening studies have revealed a number of potential prostate cancer-specific markers, but their diagnostic feasibility and prognostic value remain controversial (Esfahani, Ataei & Panjehpour, 2015; Hoogland, Kweldam & Van Leenders, 2014; Mohammed, 2014; Thompson, 2006). Today the most common clinical test for prostate cancer is prostate-specific antigen (PSA) level evaluation (Thompson, 2006), but this method meets severe limitations. First, PSA is detected in about 50% of benign prostate hyperplasia cases, and PSA-negative cases of prostate cancer also occur. Second, PSA levels can be altered by different infections of the urinary tract and by various drug treatments (Barry, 2001; Thompson et al., 2006) and thus cannot be considered a reliable diagnostic marker.

The novel promising markers that fit the criteria of reliability and sensitivity have been proposed over the years (Amaro et al., 2014). Among them, a rare isoform of motor protein myosin 1C named myosin 1C isoform A (Ihnatovych et al., 2012) has been described as a specific marker of prostate cancer cells (Ihnatovych, Sielski & Hofmann, 2014). In contrast to isoforms B and C that are ubiquitously expressed in normal tissues, myosin 1C isoform A is only present at low levels in pancreas, kidney, adrenal gland and a subset of adipose tissues (Sielski et al., 2014). In cancers, specific elevation of myosin 1C isoform A expression levels were described for prostate cancer cell lines and for TRAMP murine model that closely resembles the human prostate cancer pathogenesis (Shappell et al., 2004). Most recently, this isoform was found to have a function in the motility and secretion stimulating the invasive properties of metastatic prostate cancer cells (Maly et al., 2017) These data strongly support a role of myosin 1C isoform A as a putative diagnostic marker for prostate cancer.

One of the most time and cost-effective approaches to myosin 1C isoform A relative expression assessment in cancer and non-cancer specimens is quantitative polymerase chain reaction (RT-qPCR). Accurate normalization of qPCR data is a prerequisite that allows obtaining reliable quantitative data, while biased normalization may yield inaccurate results (Dheda et al., 2005). Several approaches to reference gene choice had been proposed over the years (Pfaffl et al., 2004; Vandesompele et al., 2002) have significantly improved data analysis for clinical sample sets (Potashnikova, Gladkikh & Vorobjev, 2015; Schmidt et al., 2006; Wotschofsky et al., 2011). Various combinations of reference genes, including *ACTB, GAPDH, ALAS-1, HPRT1, RPL13A, SDHA, TBP*, and *B2M* were evaluated for qPCR data normalization on prostate tissue samples and cell lines (Mori et al., 2008; Tsaur et al., 2013; Zhao et al., 2018). As a universal combination of reference controls for all targets and experimental conditions does not exist (Vandesompele et al., 2002), picking the cell- or tissue-specific set of genes is an essential step for any individual application.

In this study, we validate the optimal combination of reference genes for myosin 1C isoform A expression assessment in both normal and cancer prostate cells and define a limit of detection for myosin 1C isoform A mRNA expression using RTqPCR. Based on the literature, we selected from the frequently used housekeeping genes a set for quantitative analysis of myosin 1C isoform A expression in 3 prostate cancer lines (LNCaP, 22Rv1, and PC3) and RWPE-1 cell line corresponding to benign adult human prostate. Sequentially, we defined a limit of detection of myosin 1C isoform A mRNA in a mixture of cancer and non-cancer prostate cells and proved the specificity of myosin 1C isoform A expression at protein level. Our data strongly support a hypothesis of myosin 1C isoform A being a specific and sensitive marker of prostate cancer and provide a reliable and cost-effective method of myosin 1C isoform A assessment in malignant and benign prostate cells.

## Material and Methods

### Cell lines

One normal prostate epithelial cell line (RWPE-1), prostate cancer cell lines LNCaP, 22Rv1 and PC3, A431 epidermoid carcinoma, A549 lung carcinoma, and non-cancer 3T3 fibroblasts were obtained from American Type Culture Collection (ATCC, http://www.atcc.org, Manassas, VA, USA). RWPE-1 cells were cultured in DMEM (Paneco, Russia), with 4.5 g/L glucose, containing 10% FBS (Gibco, Carlsbad, CA, USA), 1% gentamycin and supplemented with 0.05 mg/ml bovine pituitary extract (BPE) and 5 ng/ml human recombinant epidermal growth factor (EGF). Cancer cell lines and 3T3 fibroblasts were cultured in DMEM/F12 (Paneco, Russia) supplemented with 10% of FBS, 1% gentamycin and L-glutamine. Cells were grown in a humidified atmosphere with 5% CO_2_ at 37 °C.

### RNA extraction and cDNA synthesis

Total RNA extraction was performed with RNeasy Mini Kit (Qiagen, Valencia, CA, USA) according to the manufacturer’s instructions, RNA samples were treated with DNAse I (Fermentas). RNA concentrations were measured on Nanophotometer (Implen, Germany), and RNA purity was confirmed by A260/280 and 260/230 ratios. 1 µg of total RNA was taken into the reverse transcription reaction with iScript Advanced cDNA synthesis kit (Bio-Rad, Hercules, CA, USA) according to manufacturer’s protocol.

### RT qPCR

Real-time qPCR experiments were performed on CFX96 Touch cycler (Bio-Rad, Hercules, CA, USA). All samples were processed in triplicate. One sample of cDNA put into each PCR run served as an inter-run calibrator for uniting data into one experiment. Primer details are provided in Table S1. All amplicon sequences included at least one exon-exon junction to avoid DNA amplification. Primers were purchased from Synthol (Russia). Primer specificity was confirmed by melting curve analysis and detection of products with predicted length on 1.5% agarose gel electrophoresis. Amplification efficiency *E* was calculated as *E* = [10(−1∕slope) − 1], using the slope of the semi-log regression plot of Cq versus log input of cDNA. Each reaction was performed in triplicate. The reaction and cycling conditions were performed as described elsewhere (Potashnikova, Gladkikh & Vorobjev, 2015).

The *Ct* values were determined for real-time PCR curves by setting the threshold at 5 SD for each run. Relative cDNA quantity was calculated as

\begin{eqnarray*}\mathbf{Q}={\mathbf{E}}^{c(q)\min \nolimits -C(q)n}, \end{eqnarray*}

where E—PCR efficiency, *C*(*q*)*n*—averaged triplicate *C*(*q*) value for each sample, *C*(*q*)min—minimal average *C*(*q*) value for the gene in the experimental set.

Results were analyzed using geNorm (Center of Medical Genetics, Ghent University hospital, geNorm version 3.5, 2002) and NormFinder (Molecular Diagnostic Laboratory, Department of Clinical Biochemistry, Aarhus University Hospital, Denmark, 2004).

### Immunofluorescence

Mouse monoclonal antibody against N-terminal peptide of myosin 1C isoform A was described in Ihnatovych et al. (2012) (Ihnatovych et al., 2012). For myosin 1C isoform A visualization, cells were fixed with 4% paraformaldehyde in PBS, pH 7.2, for 15 min at 4 °C, washed three times with PBS, permeabilized with 0.1% Triton X100 and 0.1% Tween 20 in PBS for 1 h at room temperature, stained with first antibodies (1:150 dilution), then with second anti-mouse antibodies conjugated with Cy2 (Sigma-Aldrich, St. Louis, MO, USA) (dilution 1:100) and DAPI (Sigma-Aldrich, St. Louis, MO, USA) for nucleus visualization. Specimens were mounted in Mowiol 488 (Sigma-Aldrich, St. Louis, MO, USA). Fixed cells were imaged on Nikon TiE fluorescent microscope under PlanApo 60 ×/1.4 objective (phase contrast). Images were recorded by Hamamatsu ORCA FLASH2 digital camera, using FITC (Ex. 450–490 nm; Em. 510–540 nm) and DAPI (Ex. 340–380 nm; Em. 435–485 nm) filter sets. Preparation conditions and exposure time was kept constant for all specimens used.

### Image analysis

Data were analyzed using ImageJ software (NIH, Bethesda, MD, USA). Quantitative measurements were performed on the unprocessed original 16-bit B/W images. To perform the analysis of mean fluorescence intensity values, we picked the small image regions of the same area inside the nucleus, in the cytoplasm and outside the cell (these values were taken for the signal-to-noise ratio), calculated the average fluorescence intensity values and performed signal to noise correction. Fluorescent images were processed using ImageJ and finalized with Adobe Photoshop (Adobe Systems, San Jose, CA, USA) software.

### Flow cytometry

PC3 and RWPE-1 cells (at approx. 50% confluence) were detached using trypsin then consecutively washed with 10% FBS-medium and PBS. Detached cells were stained for flow cytometry in PBS according to antibody manufacturers’ protocols (15 min at +4 °C, in the dark). Antibody details were as follows: anti-CD44-BV421 (clone BJ10, BioLegend), anti-CD90-BV510 (clone 5E10, BioLegend), anti-CD133/1-PE (clone AC133, Miltenyi Biotec), anti-CD57-PerCp-Cy5.5 (clone HNK-1, BioLegend), anti-CD24-APC-H7 (clone ML5, BD). Cells were washed with PBS and analyzed using a 6-laser FACSAria SORP instrument (BD Biosciences, USA).

Median fluorescence intensity (MFI) values were obtained from FACSDiva 6.1.3 software for each surface marker under study in the overall PC3 and RWPE-1 cell populations. The overall cell populations were gated based on light scatter properties and median signal intensities were exported for them. Immunophenotypes of the model cell lines were monitored several times throughout cell passaging. Average MFIs and their SDs were calculated for surface markers from different passage data.

### Data analysis

Statistical data and graphs were obtained using GraphPad^®^ Prism (GraphPad Software, version 5; San Diego, CA, USA). Data were presented as medians and ranges or as median ± SEM. Differences between data sets were tested with the Mann–Whitney *U* test, inter-group differences with *p* < 0.05 were reported as significant.

## Results

### Immunophenotype of prostate cell lines

**Figure 1:**
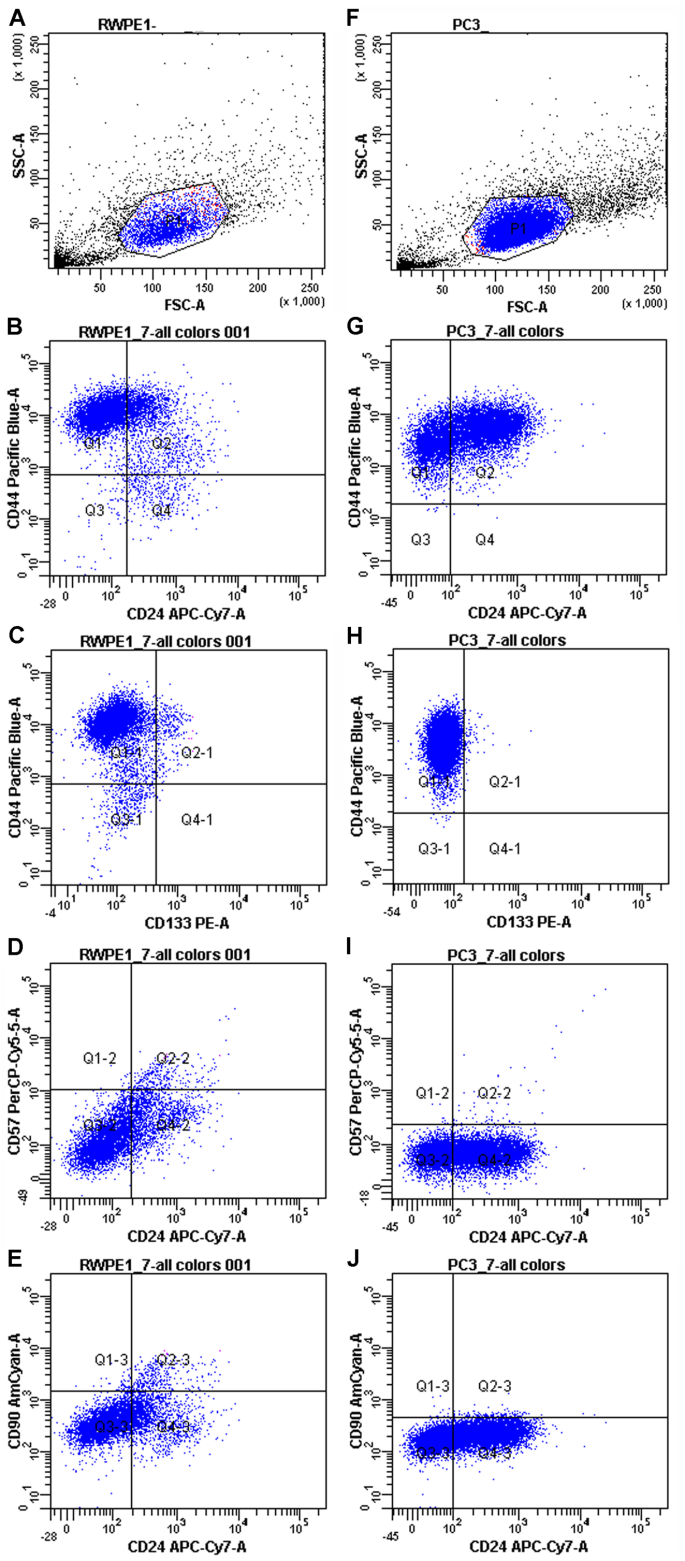
Immunophenotypes of the model cell cultures RWPE-1 (A–E) and PC3 (F–J) subcultured under passage 8.

First, PC3 and RWPE-1 model cell lines were immunophenotyped by flow cytometry. Both cell lines are characterized as CD44+/CD90-/CD133-/CD57-/CD24+- when subcultured under passage 8 (Fig. 1).

However, some markers (CD44 and CD24) showed high MFI (Mean fluorescence intensities) variance between passages in both PC3 and RWPE-1 cell lines. This can be best illustrated by the temporal heterogeneity of CD44 expression during RWPE-1 early passages (Fig. S1): here we show an increase in a stem cell marker CD44 expression upon passage 4. A minor CD133-positive subpopulation appears at passage 8, while CD90 remains absent at all passages.

To access the MFI variance, average MFI values for each marker and their SDs were calculated for both cell lines based on three immunophenotypes from different passages. For PC3 cells they were as follows: CD44 MFI = 9489.67 ± 8605.98, CD90 MFI = 172 ± 43.35, CD133 MFI = 101.67 ± 34.59, CD57 MFI = 78.67 ± 12.58, CD24 MFI = 136.50 ± 57.28. The average MFI values for RWPE-1 cells were as follows: CD44 MFI = 9567.67 ± 7871.92, CD90 MFI = 176.33 ± 95.03, CD133 MFI = 137.67 ± 37.63, CD57 MFI = 121.67 ± 32.32, CD24 MFI = 142.00 ± 50.91.

Both cancer and non-cancer prostate cell lines exhibited the same set of surface markers, thus flow cytometry as a sole method was not enough to distinguish prostate cancer cells. For better evaluation of the differences between cancer and non-cancer cells we employed RT qPCR.

### Optimal reference genes for prostate cell lines

The choice of external reference controls for normalization and qPCR data analysis is critical in case of low mRNA quantities. Reference genes have to be expressed ubiquitously in normal and malignant tissue, and their expression should not be affected by experimental conditions. The reliable set of reference genes has to include high abundance genes (that correspond to transcriptional levels of *MYO1C A* isoform in cancer cells) and low abundance genes (that correspond to transcriptional levels of *MYO1C A* isoform in normal prostate). A sufficient number of genes for qPCR data normalization also have to be estimated.

Initial screening confirmed that all candidate genes were expressed in prostate cell lines. Raw Cq values of the chosen genes indicate that the list of candidates includes genes with high (*HPRT1)*, moderate *(ACTB, TBP, YWHAZ, RPL13A, B2M, ALAS1*) and low (*UBC, SDHA, GAPDH)* abundance (Fig. S2). qPCR analysis was verified by the presence of a single sharp peak on the melting curve and a single product band of predicted size on agarose gel electrophoresis. For all candidate genes, efficiency values ranged between 0.93 and 1.05 and *R*^2^ values ranged between 0.992 and 0.999 (Table S2). We also evaluated the expression of the candidate reference genes for the cells on different passages and showed that mRNA expression of candidate genes in RWPE-1 and PC-3 lines did not change at least for 10 passages, as standard deviation of the normalized cDNA quantity for all genes did not exceed 0.04 (Table S3). Expression stability of the selected genes was determined with GeNorm and NormFinder algorithms.

In GeNorm, we ranked the selected genes according to their expression stability (*M* value). All candidate genes except for *B2M* and *RPL13A* passed the 1.5 stability threshold value proposed by GeNorm. GeNorm ranking order indicated that *YWHAZ/GAPDH* pair had the lowest *M* value (0.52). *HPRT1* and *ACTB* genes also had stability values below 1 (Table 1). Unlike GeNorm, NormFinder does not evaluate paired variances and estimates the independent stability values (SV). NormFinder ranking established *GAPDH* as the most stable single gene across all cell lines under study (SV = 0.08) and *HPRT1* as the second stable gene with SV = 0.11, while *YWHAZ* had SV = 0.13 (Table 1).

**Table 1:** Ranking order and best reference genes defined by GeNorm and NormFinder.

GeNorm	*M* value	NormFinder	SV (stability value)
*YWHAZ*	0.52	*GAPDH*	0.08
*GAPDH*	0.52	*HPRT1*	0.11
*HPRT1*	0.75	*YWHAZ*	0.13
*ACTB*	0.78	*ACTB*	0.27
*TBP*	1.02	*SDHA*	0.30
*UBC*	1.08	*TBP*	0.32
*SDHA*	1.22	*UBC*	0.44
*ALAS1*	1.38	*B2M*	0.54
*B2M*	1.75	*ALAS1*	0.58
*RPL13A*	1.98	*RPL13A*	0.61

**Figure 2:**
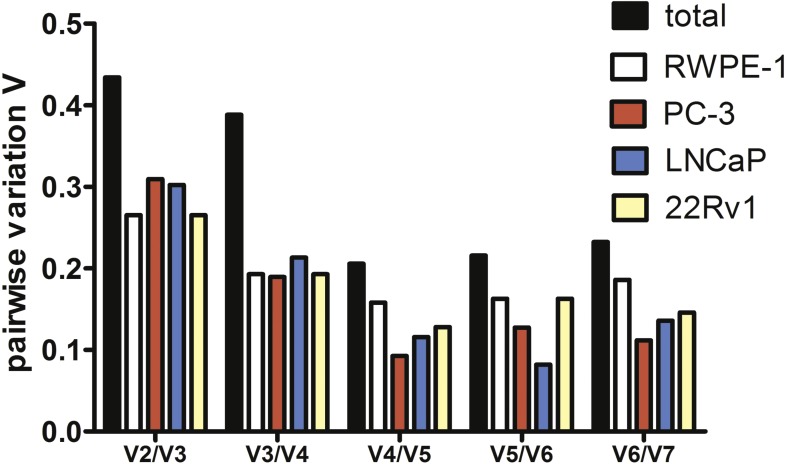
Pairwise variation analysis (*Vn*∕*n* + 1) in GeNorm program to determine the optimal number of reference genes for qPCR data normalization in prostate cancer cells.

To estimate the optimal number of genes for qPCR data normalization we employed pairwise variation analysis proposed by GeNorm. Pairwise variations are defined as SD of log2-transformed expression ratios of any pair of reference genes required for geometric mean normalization. As shown in Fig. 2, the lowest *V* values were for V4/V5 for all cell lines except LNCaP, V4/V5 was also the lowest for the total set, indicating that addition of the fourth gene to normalization factor (NF) is unnecessary. Put together, our data imply that a combination of *YWHAZ, GAPDH*, and *HPRT1* serves best for mRNA expression analysis in the set of prostate cell lines.

### mRNA levels of myosin 1C isoform A in malignant and benign prostate cell lines

**Figure 3:**
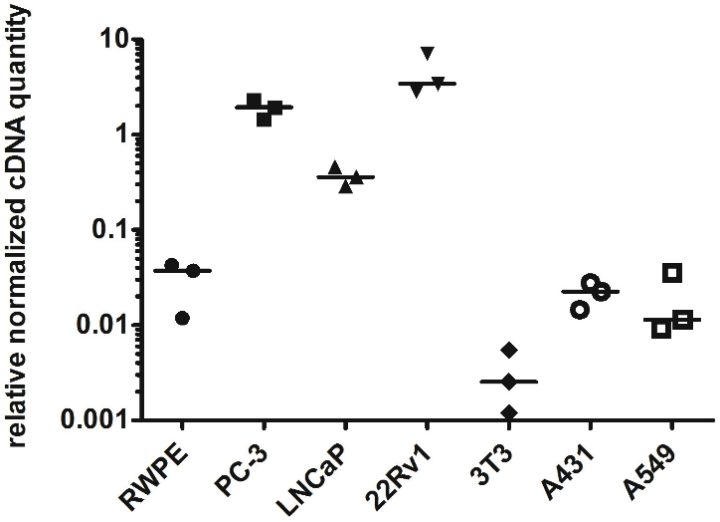
Normalized cDNA quantity of myosin 1C isoform A in cancer and non-cancer cell lines, lines on the plot represent medians.

To prove the prostate cancer-associated expression of myosin 1C isoform A we analyzed its expression in prostate cancer cell lines (PC3, LNCaP, and 22Rv1) and compared it to non-cancer prostate cell line RWPE-1. We confirmed the previous data of Ihnatovych, Sielski & Hofmann (2014) and showed that relative normalized cDNA quantity of myosin 1C isoform A in prostate cancer cells is at least two orders of magnitude higher than in non-cancer prostate cells. Medians of relative normalized cDNA quantity in prostate cancer cell lines varied from 1.938 (PC3) to 3.426 (22Rv1) compared to 0.037 in RWPE-1 cells. Myosin 1C isoform A was not expressed in normal fibroblasts (3T3 cell line, median normalized cDNA quantity 0.003) and demonstrated low expression levels in non-prostate cancers, like A431 epidermoid carcinoma (median mRNA expression 0.022) or A549 human lung carcinoma (median mRNA expression 0.011) (Fig. 3, Table 2). Myosin 1C A isoform expression for PC3 and RWPE-1 did not change significantly for different passages; relative normalised quantity of myosin 1C isoform a on the 10th passge was 2.012 for PC3 and 0.045 for RWPE-1, respectively.

**Table 2:** Normalized relative cDNA quantity of myosin 1C A isoform in different cell lines.

	RWPE-1	PC-3	LNCaP	22Rv1	3T3	A431	A549
Number of values	3	3	3	3	3	3	3
Minimum	0.01185	1.454	0.2891	2.855	0.0012	0.01455	0.009244
25% percentile	0.01185	1.454	0.2891	2.855	0.0012	0.01455	0.009244
Median	0.03716	1.938	0.3597	3.426	0.00254	0.02255	0.01146
75% percentile	0.0427	2.293	0.4567	7.078	0.005478	0.02766	0.03542
Maximum	0.0427	2.293	0.4567	7.078	0.005478	0.02766	0.03542

**Figure 4:**
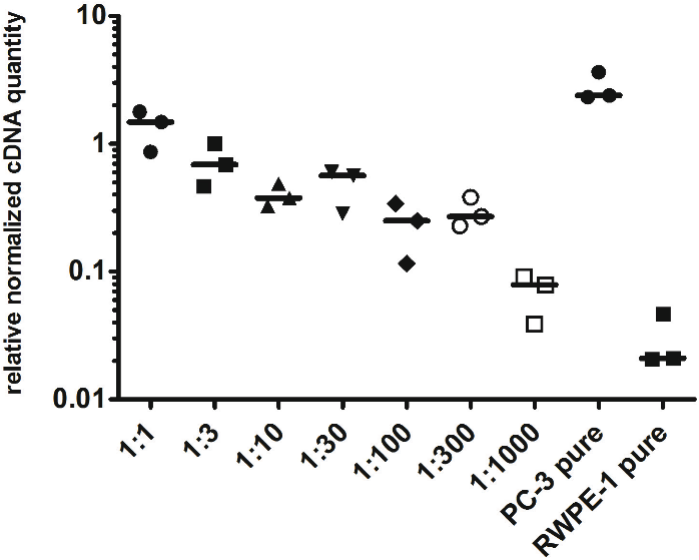
Normalized cDNA quantity of myosin 1C isoform A in series of PC3 and RWPE-1 mixtures. lines on the plot represent medians.

To evaluate the detection limit of myosin 1C isoform A with real-time qPCR, we performed a series of dilutions of PC3 cells by RWPE-1 cells as follows: 1:1, 1:3, 1:10, 1:30, 1:100, 1:300 and 1:1,000 (where one part corresponded to 10^4^ PC3 cells). Normalized relative cDNA quantity of myosin 1C isoform A varied from 1.478 (1:1 mixture) to 0.078 (1:1,000 mixture) compared to 2.389 for pure PC3 cells and 0.021 for pure RWPE-1 cells (Fig. 4). Thus, qPCR evaluation of myosin 1C isoform A expression can be considered a sensitive and selective method for prostate cancer detection, even for low quantities of cancer cells in the specimen.

### Protein expression of myosin 1C isoform A is prostate cancer-specific

**Figure 5:**
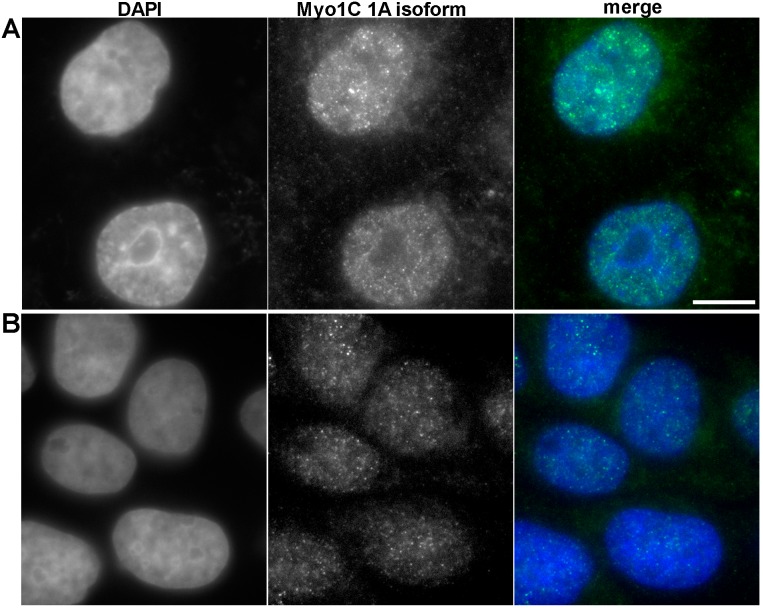
Immunofluorescent anti myosin 1C isoform A staining of PC3 (A) and RWPE-1 (B) cells.

To confirm that myosin 1C isoform A is a specific marker of prostate cancer at protein level, we performed immunofluorescence staining of PC3 and RWPE-1 cells using specific antibodies against myosin 1C isoform A. In PC3 cells we observed the bright staining of myosin 1C isoform A throughout the cytoplasm and in the nucleus (Fig. 5A), whereas in RWPE-1 cells the staining was much weaker, although the protein was also located both in the nucleus and in the cytoplasm (Fig. 5B). Further staining of A549 cells with the antibody against myosin 1C isoform A also revealed a weak signal both in the nucleus and in the cytoplasm (Fig. S3).

For quantitative evaluation of protein expression, we performed mean fluorescence intensity calculations for myosin 1C isoform A staining in the nucleus and cytoplasm on 58 PC3 cells and 56 RWPE-1 cells. Median MFI of myosin 1C isoform A staining for nuclei of PC3 cells was 1749.0 compared to 924.9 in RWPE-1 cells (differences were statistically significant, *p* < 0.0001). The differences in cytoplasmic staining of myosin 1C isoform A were less distinct, median MFI of the cytoplasmic signal was 813.5 for PC3 cells and 602.1 for RWPE-1 cells (differences were statistically significant, *p* < 0.0001) (Fig. S4).

## Discussion

The significance of surface cluster of differentiation (CD) markers in establishing cell origins and cancer-associated aberrations in prostate tissue is well-known. Markers (such as CD133, CD44, CD90, CD57 and CD24, etc.) are being widely used in diagnostics by flow cytometry and immunohistochemistry (Liu, Roudier & True, 2004). In our study CD90 was used as a stromal marker (for both normal and malignant prostate tissue (Liu, Roudier & True, 2004; True et al., 2010; Zhao & Peehl, 2009), CD57—as luminal cell marker (Liu, Roudier & True, 2004), CD44 and CD133—as epithelial basal stem cell markers (Iczkowski, 2010; Richardson et al., 2004), and CD24—as prostate epithelial cell maturation marker (Petkova et al., 2013).

The model prostate cancer cell line PC3 used in our study proved to be CD44+ and CD133-, as shown by other authors (Pellacani et al., 2011; Steponkiene et al., 2011). It also is CD90- and CD57- in accord with other published data (Liu, 2000; Liu, Roudier & True, 2004). The data on CD24 expression are controversial as some authors report no CD24 on PC3 cells (Liu, Roudier & True, 2004) and some show varying levels of this surface marker (Jaworska & Szliszka, 2017; Liu, 2000). Our instance of PC3 cell line also was partly positive for CD24 and its level varied at different passages (see ‘Results’).

The model benign prostate cell line RWPE-1 used in our study exhibited the CD44+/CD90-/CD57-/CD133-/CD24+- immunophenotype similar to PC3 cell line. It demonstrates gradual elevation of CD44 level after passage 3 and a small subpopulation of CD133+ positive RWPE-1 cells appears at passage 8. This corresponds to the data published by other authors (Yun et al., 2016), and means that this cell line should be cautiously used as a reference one.

The overall phenotype of both PC3 and RWPE-1 cell lines provided in Fig. 1 defines them as low-differentiated (basal –to transitional) cells of epithelial origin. CD44 shows the highest temporal expression heterogeneity, depending on passage number while CD24 shows the highest intra-population expression heterogeneity in both cell lines. The obtained data highlights the problem of immunophenotype shifts in putative prostate cell models (both normal and malignant) upon prolonged subculture. Other properties of such models may also vary and thus need to be monitored during subculture.

While the morphological phenotype and functional properties of RWPE-1 cells correspond to normal prostate tissue, its immunophenotype varies from early passages and final immunophenotypes upon prolonged passaging become similar in normal and malignant cell models (Bello et al., 1997; Jiang et al., 2010; Roh et al., 2008). Hence, the need for other markers to correlate with tumorigenic potential of prostate cells is obvious. We propose to employ qPCR assessment of the novel cancer-related molecules once their correlation with protein levels has been established.

PCR data normalization using a set of reference genes and different mathematical algorithms has proved to be a valuable methodology in different types of cancer, including bladder cancer (Zhang et al., 2017), hepatocellular carcinoma (Liu et al., 2013), uterine cervical cancer (Tan et al., 2017) and cancer cell lines used for *in vitro* studies (Jacob et al., 2013). In all cases one has to follow the MIQE guidelines that describe the minimum information for publication of PCR data to provide the consistency between the laboratories and reproducibility of clinical and scientific results (Bustin et al., 2009).

The choice of reliable reference genes remains a challenging issue even within one tissue, as it depends on the cell source, application type, and sample heterogeneity. Souza et al. (2013) proposed *GAPDH* and *SDHA* genes as an optimal combination for RT qPCR data normalization in primary culture of prostate cells, Mori et al. (2008) suggested *GAPDH* and *ACTB* as suitable controls for FFPE-embedded prostate tissue, while *HPRT1* and *TBP* were the most appropriate genes for prostate cancer metastases into the lymph nodes (Tsaur et al., 2013), thus confirming a prerequisite analysis of external reference control prior to evaluation of target gene in prostate cancer cells.

Here we report that a combination of three, but not two genes represents the best combination for normalization using geometric mean even in homogeneous cell line samples. Using a set of three reference genes (*YWHAZ, GAPDH*, and *HPRT1*), we analyzed myosin 1C isoform A mRNA expression in a set of cancer and non-cancer cell lines and confirmed the specificity of its expression in prostate cancer cells. Interestingly, the median expression of myosin 1C isoform A in LNCaP cells was almost six times lower than in PC3 cells (Table 2). One possible explanation might be that LNCaP cells are derived from androgen-dependent prostatic adenocarcinoma (Horoszewicz et al., 1980; Yu et al., 2017) in contrast to androgen-independent PC3 and 22Rv1 prostate carcinomas. Nevertheless, these cell lines have many other differences including the derivation site (Horoszewicz et al., 1980; Kaighn et al., 1979), genomic abnormalilties (Seim et al., 2017) and gene expression profile (Ravenna et al., 2014), the intrinsic reason of Myosin 1C isoform A downregulation in LNCaP cells and practical implementation of this finding has to be further evaluated. Thus, myosin 1C isoform A expression should be further evaluated in a set of clinical samples of androgen-dependent and androgen-independent prostate cancers. However, myosin 1C isoform A mRNA is a sensitive and reliable marker of cancer prostate cells, as it allows to detect even 10^4^ tumor cells in a total mixture of 10^7^ cells. While the difference in mRNA expression of myosin 1C isoform A between PC3 and RWPE-1 cells was almost two orders of magnitude (about 50 times), a robust evaluation of differences in protein expression revealed that MFI intensity of myosin 1C isoform A (which can be used as a surrogate comparative value for evaluation of protein expression) in PC3 nuclei is almost two times higher than in RWPE-1 cells, while differences in MFI for cytoplasmic signal for the same cells was only 1.35 times. Notably, nucleus/cytoplasm MFI ratio was higher in PC3 cancer prostate cells compared to RWPE-1 cells (2.14 vs. 1.53) consistent with the previous data on predominantly nuclear localization of myosin 1C isoform A (Ihnatovych et al., 2012). Ihnatovych, Sielski & Hofmann (2014) showed that the difference in myosin 1C isoform A protein expression between PC3 and RWPE-1 cells was almost eight times using the semi-quantitative analysis of Western blot data. An established role of regulatory processes for protein and mRNA expression (Vogel & Marcotte, 2012) can explain the absence of the direct correlation for mRNA and protein level for myosin 1C A isoform, as the half-life time and structural modifications of this protein have to be further elucidated. Single cell analysis with RNA probes and antibodies could give a more specific insight into this issue and can be regarded as a future perspective for this field.

Taken together, these data confirm the specificity of myosin 1C isoform A expression in prostate cancer cells both on mRNA and protein level and strongly suggest RT qPCR as a method of choice for evaluation of myosin 1C isoform A expression on a set of prostate tissue samples.

##  Supplemental Information

10.7717/peerj.5970/supp-1 Supplemental Information 1 Supplementary figures and tables

10.7717/peerj.5970/supp-2 Supplemental Information 2 Raw data for threshold cycles and MFI values

10.7717/peerj.5970/supp-3 Supplemental Information 3 Changes in immunophenotype of RWPE1 cell line upon passaging

10.7717/peerj.5970/supp-4 Supplemental Information 4 Box plots for raw Cq values that represent mRNA expression for each candidate reference gene

10.7717/peerj.5970/supp-5 Supplemental Information 5 Immunofluorescent anti-myosin 1C isoform A staining of A-549 cells, bar 10 μm

10.7717/peerj.5970/supp-6 Supplemental Information 6 MFI values for myosin 1C isoform A fluorescence intensity in nuclei and cytoplasm of PC3 and RWPE-1 cells

## Additional Information and Declarations

### Data Availability

The following information was supplied regarding data availability: The raw data are provided in Supplementary File 2.
