# Supplementary material for: Specific and reliable detection of Myosin 1C isoform A by RTqPCR in prostate cancer cells
Source: PeerJ. 2018 Nov 20;6:e5970. doi: 10.7717/peerj.5970 (PMC6251347; doi:10.7717/peerj.5970)
Supplement: Supplemental Information 1 [file peerj-06-5970-s001.doc]

**Supplementary figures and tables**

***Figure S1****. Changes in immunophenotype of RWPE1 cell line upon passaging. A CD44 high subpopulation appears for the first time at passage 4 and becomes dominant at passage 8.*

**
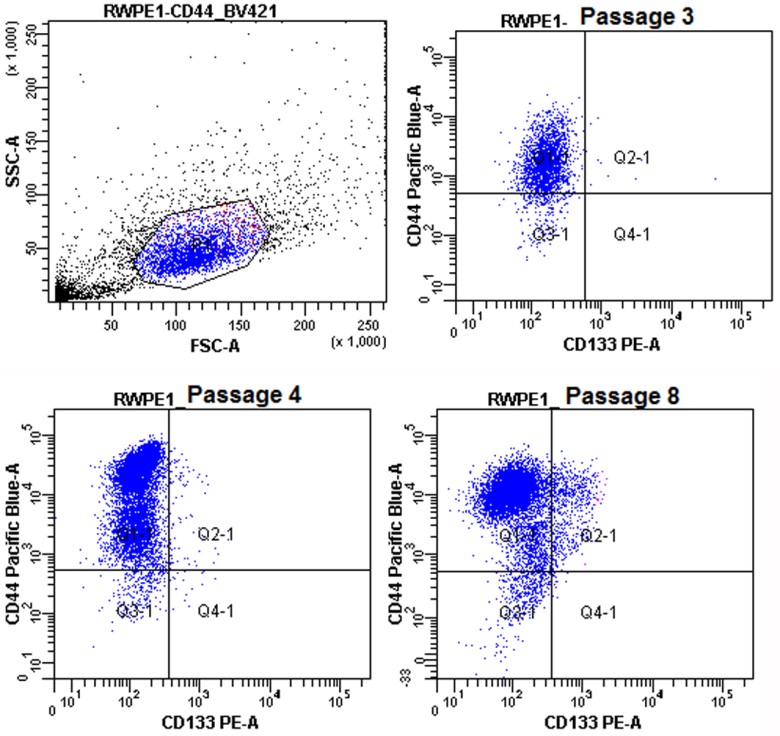
**

***Figure S2.*** *Box plots for raw Cq values that represent mRNA expression for each candidate reference gene. Data set of raw Cq was collected for all prostate cell lines. Median is indicated as a line inside the box, which in turn represents the 25th and 75th percentile. Whiskers indicate the 10/90 percentile range.*


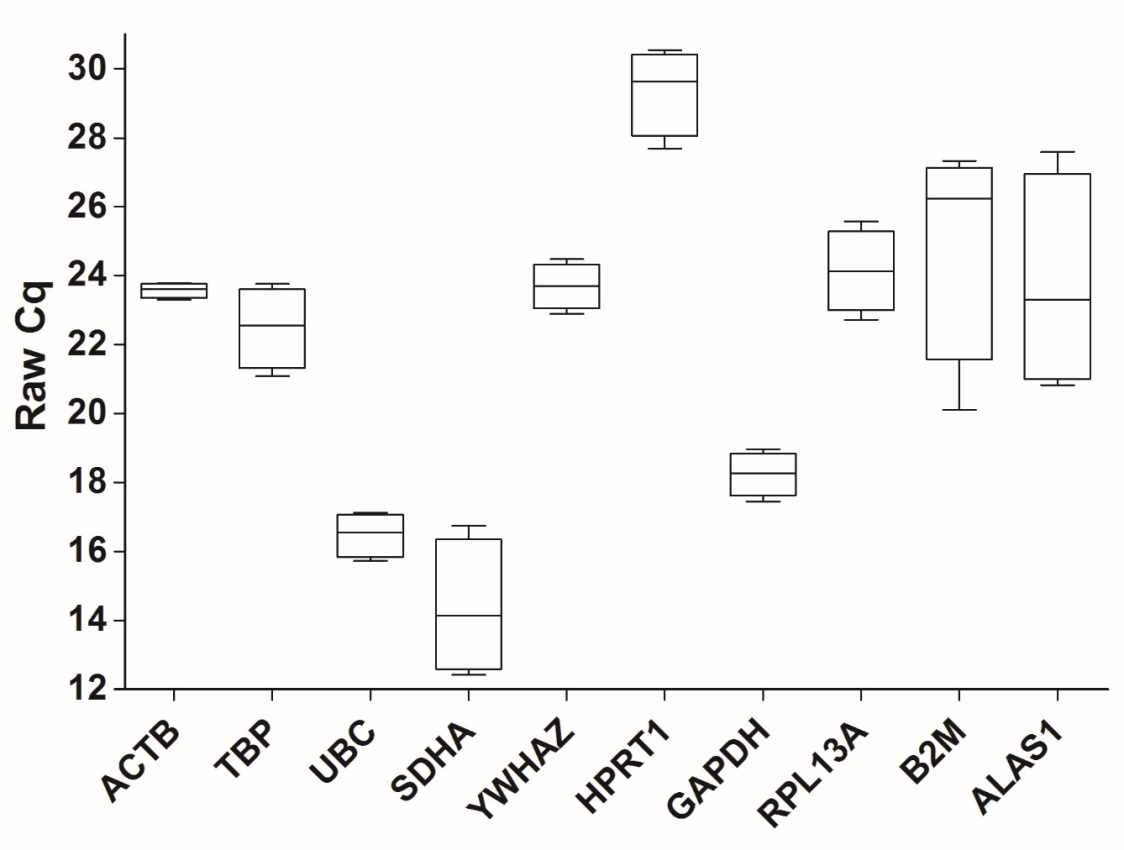


***Figure S3.*** *Immunofluorescent anti-myosin 1C isoform A staining of A-549 cells, bar 10 µm*

**
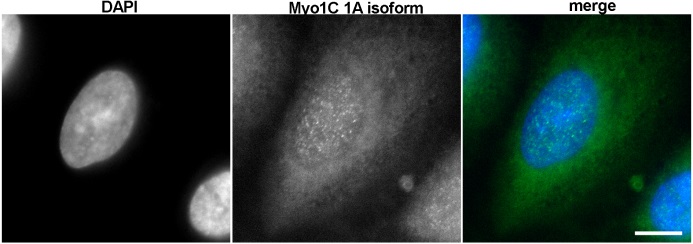
**

***Figure S4.*** *MFI values for myosin 1C isoform A fluorescence intensity in nuclei and cytoplasm of PC3 and RWPE-1 cells. Data are given as median±SEM.*

**
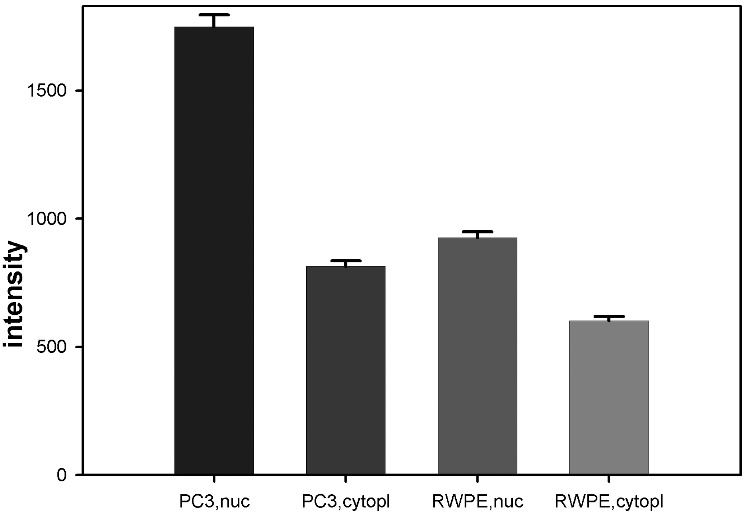
**

**Table S1. Primer sequences for RT qPCR mRNA evaluation**

| Gene symbol | Forward and reverse primer sequence | Amplicon size | Localization | Reference |
| --- | --- | --- | --- | --- |
| *YWHAZ* | ACTTTTGGTACATTGTGGCTTCAA  CCGCCAGGACAAACCAGTAT | 94 | 2p25 | 14 |
| *HPRT1* | TGACACTGGCAAAACAATGCA  GGTCCTTTTCACCAGCAAGCT | 94 | Xq26 | 14 |
| *UBC* | ATTTGGGTCGCAGTTCTTG  TGCCTTGACATTCTCGATGGT | 133 | 12q24 | 14 |
| *GAPDH* | TGCACCACCAACTGCTTAGC  GGCATGGACTGTGGTCATGAG | 87 | 12p13 | 14 |
| *ACTB* | CTGGAACGGTGAAGGTGACA  AAGGGACTTCCTGTAACAATGCA | 140 | 7p15-p12 | 14 |
| *RPL13A* | CCTGGAGGAGAAGAGGAAAGAGA  TTGAGGACCTCTGTGTATTTGTCAA | 126 | 19q13 | 14 |
| *B2M* | TGCTGTCTCCATGTTTGATGTATCT  TCTCTGCTCCCCACCTCTAAGT | 86 | 15q21-q22 | 14 |
| *TBP* | GAGAGTTCTGGGATTGTACCG  ATCCTCATGATTACCGCAGC | 143 | 6q27 | 20 |
| *ALAS1* | AGTGTGAAAACCGATGGAGG  CGATCATACTGAAAAGTGGAAACAG | 140 | 3p21 | 20 |
| *SDHA* | TGGTTGTCTTTGGTCGGG  GCGTTTGGTTTAATTGGAGGG | 85 | 5p15 | 20 |
| *MYOIC (isoform A)* | GGAGAGATCATCCGTGTGGT  GGACCGATGTAGGTATAAAGAGG | 215 | 17p13 | 11 |

**Table S2**. qPCR efficiency for candidate reference genes, R^2^ is correlation coefficient.

| Gene | R^2^ | slope | Efficiency |
| --- | --- | --- | --- |
| *ACTB* | 0.994 | -3.342 | 0.99 |
| *TBP* | 0.999 | -3.493 | 0.93 |
| *UBC* | 0.974 | -3.419 | 0.96 |
| *SDHA* | 0.989 | -3.382 | 0.97 |
| *YWHAZ* | 0.996 | -3.280 | 1.02 |
| *HPRT1* | 0.965 | -3.374 | 0.98 |
| *GAPDH* | 0.999 | -3.212 | 1.05 |
| *RPL13A* | 0.992 | -3.291 | 1.01 |
| *B2M* | 0.998 | -3.507 | 0.93 |
| *ALAS1* | 0.996 | -3.418 | 0.96 |

Table S3. mRNA expression of the candidate genes on the passages 1, 5 and 10. Data are given as related normalized cDNA quantity, SD is standard deviation for mRNA expression of the candidate gene between 3 passages.

| RWPE-1 |  |  |  |  | PC-3 |  |  |  |  |
| --- | --- | --- | --- | --- | --- | --- | --- | --- | --- |
|  | Passage 1 | Passage 5 | Passage 10 | **SD** |  | Passage 1 | Passage 5 | Passage 10 | **SD** |
| *ACTB* | 0,14453 | 0,14677 | 0,19162 | **0,02656** | *ACTB* | 0,12454 | 0,13542 | 0,19162 | **0,036001** |
| *TBP* | 0,34242 | 0,35713 | 0,28817 | **0,03632** | *TBP* | 0,40255 | 0,34575 | 0,32513 | **0,040094** |
| *UBC* | 0,32498 | 0,33103 | 0,36803 | **0,02330** | *UBC* | 0,41257 | 0,37841 | 0,31427 | **0,049906** |
| *SDHA* | 0,23136 | 0,18521 | 0,25979 | **0,03763** | *SDHA* | 0,19274 | 0,22165 | 0,17542 | **0,023356** |
| *YWHAZ* | 0,10747 | 0,07314 | 0,07235 | **0,02005** | *YWHAZ* | 0,11475 | 0,08214 | 0,11836 | **0,019951** |
| *HPRT1* | 0,11624 | 0,13344 | 0,12863 | **0,00887** | *HPRT1* | 0,12111 | 0,13478 | 0,14255 | **0,010854** |
| *GAPDH* | 0,25910 | 0,29628 | 0,24669 | **0,02580** | *GAPDH* | 0,19774 | 0,19625 | 0,24223 | **0,026127** |
| *RPL13A* | 0,46452 | 0,41359 | 0,43590 | **0,02552** | *RPL13A* | 0,54577 | 0,48338 | 0,58924 | **0,053211** |
| *B2M* | 0,20658 | 0,23437 | 0,22180 | **0,01391** | *B2M* | 0,24558 | 0,28995 | 0,19875 | **0,045606** |
| *ALAS1* | 0,21957 | 0,22291 | 0,28099 | **0,03453** | *ALAS1* | 0,27416 | 0,21442 | 0,22145 | **0,032651** |
