# Supplementary material for: Specific and reliable detection of Myosin 1C isoform A by RTqPCR in prostate cancer cells
Source: PeerJ. 2018 Nov 20;6:e5970. doi: 10.7717/peerj.5970 (PMC6251347; doi:10.7717/peerj.5970)
Supplement: Supplemental Information 2 [file peerj-06-5970-s002.docx]

Table 1. Raw data for Cq of the candidate reference genes

| ACTB | TBP | UBC | SDHA | YWHAZ | HPRT1 | GAPDH | RPL13A | B2M | ALAS1 |
| --- | --- | --- | --- | --- | --- | --- | --- | --- | --- |
| 23,30 | 23,76 | 16,15 | 15,17 | 24,48 | 30,54667 | 18,42 | 25,57 | 25,96 | 25,05 |
| 23,52 | 23,11 | 17,12 | 12,42 | 23,56 | 30,06667 | 18,11 | 23,85 | 20,11 | 20,82 |
| 23,79 | 22,02 | 15,72 | 13,10 | 22,89 | 27,69333 | 17,45 | 22,72 | 26,51 | 21,56 |
| 23,69 | 21,08 | 16,94 | 16,74 | 23,82 | 29,19333 | 18,96 | 24,41 | 27,33 | 27,59 |

Table 2. Raw data for mean fluorescence intensities of myosin 1C isoform A staining in nucleus and cytoplasm of PC3 and RWPE-1 cells

| PC3 nucleus | PC3 cytoplasm | RWPE nucleus | RWPE cytoplasm |
| --- | --- | --- | --- |
| 1497,994000 | 580,85200 | 1066,824000 | 701,861000 |
| 1333,578000 | 649,95300 | 1053,482000 | 596,047000 |
| 1570,478000 | 802,78400 | 1014,733000 | 652,072000 |
| 1063,441000 | 593,10700 | 949,436000 | 877,091000 |
| 1247,657000 | 640,90700 | 942,193000 | 721,469000 |
| 1260,646000 | 717,84300 | 882,214000 | 664,954000 |
| 1315,457000 | 593,53300 | 1253,680000 | 929,814000 |
| 1184,130000 | 692,91700 | 977,991000 | 751,617000 |
| 1350,176000 | 828,05300 | 1095,722000 | 819,547000 |
| 2612,671000 | 1275,57200 | 1201,378000 | 651,159000 |
| 2466,319000 | 958,32800 | 1031,193000 | 588,975000 |
| 2140,447000 | 925,65600 | 926,588000 | 633,160000 |
| 1836,120000 | 722,90000 | 1051,862000 | 791,831000 |
| 2388,557000 | 1380,83700 | 1117,170000 | 677,509000 |
| 2313,491000 | 898,36200 | 1165,947000 | 708,542000 |
| 2229,499000 | 1172,26900 | 884,531000 | 595,808000 |
| 2093,976000 | 960,40000 | 1194,209000 | 654,037000 |
| 1891,161000 | 1056,89400 | 1152,460000 | 687,137000 |
| 1968,152000 | 797,38700 | 751,731000 | 658,811000 |
| 1871,273000 | 829,72900 | 611,268000 | 556,324000 |
| 1959,436000 | 884,73600 | 865,418000 | 580,259000 |
| 1189,904000 | 742,05400 | 637,109000 | 500,344000 |
| 1339,884000 | 793,82600 | 678,137000 | 505,502000 |
| 1375,339000 | 654,57100 | 648,583000 | 668,728000 |
| 1569,567000 | 794,96400 | 923,307000 | 686,489000 |
| 2251,428000 | 753,35800 | 971,758000 | 602,761000 |
| 1831,641000 | 820,73300 | 1103,428000 | 591,098000 |
| 1740,091000 | 1005,79300 | 996,471000 | 692,851000 |
| 1629,339000 | 756,75900 | 1047,280000 | 711,470000 |
| 1612,664000 | 851,26000 | 1043,780000 | 659,936000 |
| 1934,138000 | 706,96200 | 946,142000 | 976,956000 |
| 2202,981000 | 1252,78600 | 952,890000 | 601,431000 |
| 1653,852000 | 917,28100 | 903,503000 | 575,854000 |
| 1690,379000 | 874,51400 | 825,042000 | 621,608000 |
| 2077,166000 | 688,74600 | 1074,370000 | 600,692000 |
| 1760,014000 | 769,37100 | 854,069000 | 619,372000 |
| 1605,903000 | 799,26200 | 928,948000 | 710,210000 |
| 1861,683000 | 945,25700 | 711,066000 | 510,644000 |
| 1605,951000 | 793,55700 | 701,100000 | 386,206000 |
| 2087,833000 | 834,29700 | 617,773000 | 323,280000 |
| 1943,288000 | 953,91600 | 722,257000 | 529,747000 |
| 1678,624000 | 944,94600 | 871,551000 | 543,440000 |
| 1555,519000 | 806,26200 | 986,781000 | 636,830000 |
| 1558,712000 | 821,17200 | 686,752000 | 463,760000 |
| 1699,722000 | 960,86600 | 941,229000 | 566,319000 |
| 1688,254000 | 799,09800 | 862,600000 | 715,316000 |
| 1563,478000 | 974,45200 | 758,414000 | 499,722000 |
| 1712,137000 | 848,39800 | 719,049000 | 445,340000 |
| 1645,730000 | 782,44600 | 651,122000 | 582,758000 |
| 1807,180000 | 790,41300 | 916,208000 | 528,797000 |
| 2160,848000 | 675,83300 | 851,608000 | 531,687000 |
| 1837,702000 | 603,08600 | 937,097000 | 516,940000 |
| 1757,413000 | 662,04700 | 874,364000 | 475,406000 |
| 2118,510000 | 836,06700 | 826,448000 | 478,598000 |
| 1550,726000 | 861,98800 | 679,022000 | 437,609000 |
| 2231,372000 | 769,57900 | 503,749000 | 443,456000 |
| 2225,354000 | 992,97700 |  |  |
| 1999,063000 | 925,33600 |  |  |
